# Association of Use of Rehabilitation Services With Development of Dementia Among Patients With Rheumatoid Arthritis: Analysis of Domestic Data in Taiwan

**DOI:** 10.3389/fmed.2020.00446

**Published:** 2020-08-14

**Authors:** Ming-Chi Lu, Hanoch Livneh, Chieh-Tsung Yen, Hua-Lung Huang, Miao-Chiu Lin, Shu-Wen Yen, Ning-Sheng Lai, Tzung-Yi Tsai

**Affiliations:** ^1^Division of Allergy, Immunology and Rheumatology, Dalin Tzuchi Hospital, The Buddhist Tzuchi Medical Foundation, Chiayi, Taiwan; ^2^School of Medicine, Tzu Chi University, Hualien, Taiwan; ^3^Rehabilitation Counseling Program, Portland State University, Portland, OR, United States; ^4^Department of Neurology, Dalin Tzuchi Hospital, The Buddhist Tzuchi Medical Foundation, Chiayi, Taiwan; ^5^Department of Rehabilitation, Dalin Tzuchi Hospital, The Buddhist Tzuchi Medical Foundation, Chiayi, Taiwan; ^6^Department of Nursing, Dalin Tzuchi Hospital, The Buddhist Tzuchi Medical Foundation, Chiayi, Taiwan; ^7^Department of Environmental and Occupational Health, College of Medicine, National Cheng Kung University, Tainan, Taiwan; ^8^Department of Medical Research, Dalin Tzuchi Hospital, The Buddhist Tzuchi Medical Foundation, Chiayi, Taiwan; ^9^Department of Nursing, Tzu Chi University of Science and Technology, Hualien, Taiwan

**Keywords:** rheumatoid arthritis, dementia, rehabilitation, risk, cohort study

## Abstract

**Objectives:** Rheumatoid arthritis (RA) was found to trigger the higher risk of dementia. Limited information, however, is available on whether the use of rehabilitation services (RS), an integral part of healthcare programs, can lessen dementia risk for RA subjects. The aim of this study was to determine the relationship of RS use to the development of dementia in RA patients.

**Methods:** We identified 2,927 newly diagnosed patients with RA, 20–70 years of age between 1998 and 2007, from a national health insurance database. 965 patients from this sample received RS, and 1,962 patients were designated as a control group (non-RS users). Patients were followed to the end of 2012 to identify dementia incident as the end point. Cox proportional hazards regression was performed to calculate the hazard ratio (HR) of dementia risk associated with the use of RS.

**Results:** During the study period, 388 patients with RS and 1,224 controls developed dementia, representing incidence rate of 75.46 and 115.42 per 1,000 person-years, respectively. After adjusting for potential confounders, RS was found to significantly reduce dementia risk, with the adjusted HR of 0.60 (95% confidence interval [CI] = 0.53–0.67). Those who used the high intensity of RS (≧15 courses) had the greatest benefit.

**Conclusions:** Integrating RS into the conventional treatment may reduce the sequent risk of dementia for RA patients.

## Key Points

- This is the first report from the nationwide representative database to clarify the relation between RS and dementia risk among RA patients.
- After analyses were controlled for potential confounders, the use of RS was found to significantly reduce the sequent risk of dementia, with the adjusted HR of 0.60 (95% CI = 0.53–0.67).
- This study further discovered that RA patients who used the high-intensity RS (≧15 courses) had a 62% lower risk of dementia.
- The findings of this study could guide healthcare providers to more effective treatment strategies for RA patients.

## Introduction

Rheumatoid arthritis (RA), an autoimmune inflammatory arthritis disease, principally attacks the joints and affects many bodily tissues and organs, thus causing significant burden to both patients and their families through progressive functional limitations and increased physical disability (1). These phenomena together with the accompanying negative effects often impede the work productivity of those with RA. Approximately 20–30% of affected individuals are unable to work within 3 years after RA onset (2), thus leading to the enormous emotional and socioeconomic burden for patients, their families and the healthcare system. It is estimated that the medical expenses for one RA patient in the US is ~US$20,919 per year, which was nearly three times higher than that for the general population (3).

RA not only causes enormous economic burden but also triggers other crippling illnesses. A systematic review of 15 studies found a positive association between RA and cognitive impairment, such as dementia, a commonly-reported disorder with advanced age (4). Similarly, a longitudinal study found that those with autoimmune rheumatic disease, including a history of RA, were significantly more likely than those without other autoimmune rheumatic diseases to develop dementia over a period of 12 years (5). Some scholars have concluded that the link between RA and cognitive decline may be due to inflammation in the brain (6). Further, the known high risk of cardiovascular comorbidity of patients with RA may also be implicated in the cognitive impairment (4).

Recently, rehabilitation service (RS) has been used as a strategy to restore the functional independence and improve psychophysical function in patients with RA (7, 8). One meta-analysis of six reports indicated that hydrotherapy can help improve disease activity and health status (mood and tension) for patients with RA compared to no intervention (*P* < 0.01) (9). Additionally, after reviewing 18 randomized control trials that applied a physical activity-based intervention, and adopting cognitive functioning as an outcome measure, the authors found that physical exercise positively influenced global cognitive functioning for patients affected by dementia (10). The mechanism by which RS could lessen the risk of dementia was that physical exercise, a core component of RS, can increase the blood flow and oxygen supply to the brain, as well as decrease the blood pressure and lipid levels to prevent metabolic dysfunction, all risk factors for cognitive impairment disorders (10). As such, RS may provide a double benefit to patients with RA.

Although some studies have been conducted on RS needs or utilization among patients with RA, the majority took place in Western countries, and moreover, were limited to either self-reported questionnaires or medical reviews, mainly based on small samples (9, 11–13). In Taiwan, RS comprises those services which relate to rehabilitative examination and treatment, including occupational, physical, and communicative interventions. These interventions are performed by only qualified RS therapists, regardless of when the condition occurred, and is practiced in outpatient clinics or inpatient wards. A review of previous literature indicated that most studies of RS in Taiwan have focused on RS utilization in patients with cancer, hemophilia or traumatic brain injury (14–16). As of the present, there have been no longitudinal cohort studies that focused on the relationship between RS and the development of dementia among RA patients. To narrow the gap between the research questions, we used a nationwide population-based database to compare the dementia risk among RA subjects who received or did not receive RS.

## Methods

### Data Source

The analytical data in this work were obtained from the Longitudinal Health Insurance Database (LHID), which was one of the subsets of the National Health Insurance Research Database. LHID was made up of ~1,000,000 randomly sampled people and collected the corresponding medical records from 1996 to 2012 (17). The database has been confirmed by the National Health Research Institute (NHRI) to be representative of the Taiwanese population and its data has been used in other published scientific papers (18, 19). The encrypted information protects patient privacy and allows linkage of all claims for the same patient within the database.

### Ethics

This study was conducted according to the Declaration of Helsinki and was approved by the institutional review board at Buddhist Dalin Tzu Chi Hospital. The institutional review board waived the need for informed consent (written and oral) from the patients because the database utilized in this work consists of nationwide, unidentifiable, secondary data released to the public for research purposes (No. B10004021-3).

### Study Population

**Figure 1:**
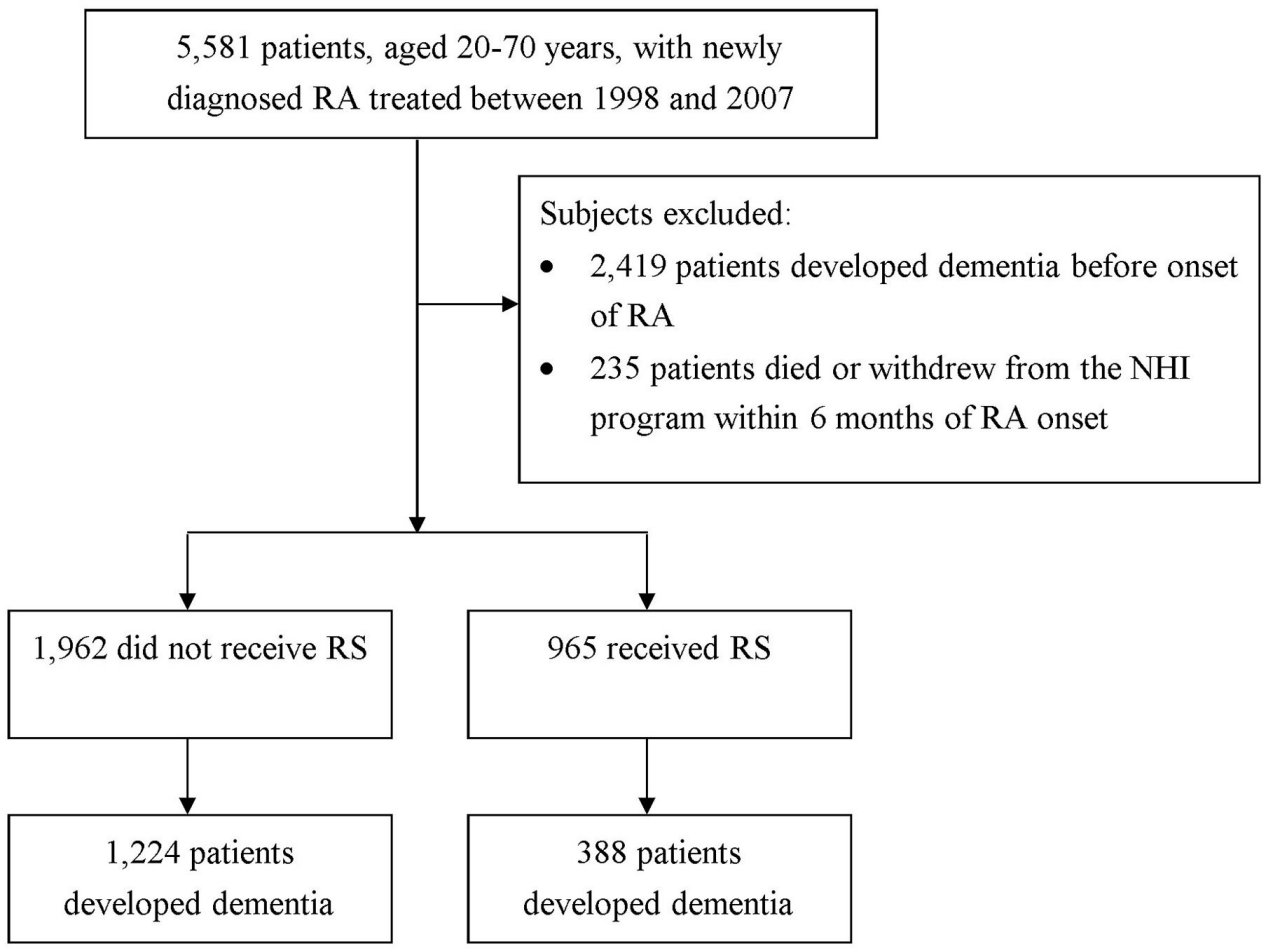
Disposition of study subjects.

In this work, to be designated as having a certain disease, the subject had to have a corresponding code by the International Classification of Disease, Ninth Revision, Clinical Modification (ICD-9-CM) in the diagnosis field. We recruited patients aged 20–70 years who sought ambulatory healthcare services for RA between 1998 and 2007 (ICD-9-CM code: 714.0). To ensure the identification of RA patients, only those patients with catastrophic illness certification due to RA were included in the study. In Taiwan, insured citizen with major diseases, such as schizophrenia, mood disorders, immune disease, and cancer, can obtain free care for their illness or related conditions within the certificate's validity period. We regarded the index date for RA patients as the date on which they gained approval for catastrophic illness registration. In order to confirm that all patients with RA in this work were incident cases, only new-onset RA cases were included (*n* = 5,581). Among them, a total of 2,419 patients diagnosed with dementia before the date of the first RA attack were excluded. The diagnostic algorithm of dementia required at least 3 outpatient visits, or at least one inpatient claim for dementia (ICD-9-CM 290, 294, or 331) during the study period. Also excluded were those with missing data and those who were not followed for a full 6 months after RA diagnosis (*n* = 235) (20). Overall, we identified 2,927 new-onset RA cases (Figure 1).

Thereafter, all subjects were linked to the healthcare claims data to determine RS usage following the onset of RA. Subjects were considered RS users if their files indicated any recorded fees for RS due to RA (14, 21). The remaining subjects were classified as non-RS users. In Taiwan, under the National Health Insurance (NHI) program, the RS include physical therapy, occupational therapy, or speech therapy. RS therapists can apply for compensation from the Bureau of NHI, and the total cost regarding RS treatment is summed up and presented in the claim database. In this study, for the RS user group, the time period between the date of RA diagnosis and the date of first RS use after RA represented the immortal time, which would induce an overestimation of the intervention's beneficial effect. To reduce this bias, we calculated person-years (PYs) starting from the commencement of RS usage to correct for immortal time for the subjects who received RS (22). The end date of the follow-up period for both groups was defined as the earliest of a diagnosis of dementia, withdraw from the insurance program, or the date of December 31, 2012. Only dementia episodes which occurred 1 year after the index date were considered in establishing a temporal link.

### Definition of Covariates

Sociodemographic factors contained age, sex, monthly incomes, and urbanization level of each patient's residential area. Monthly incomes were grouped into three levels: ≤ New Taiwan Dollar (NTD) $17,880, NTD$17,881–43,900, and ≥NTD$ 43,901. Regarding the urbanization level, it was classified into seven parts based on several dimensions, such as population density, proportion of residents with college or higher education, percentage of elderly (>65 years of age) people, proportion of the workforce in agriculture, and number of physicians per 10^5^ people (23). We further divided into three strata: urban (levels 1–2), suburban (levels 3–4) and rural (levels 5–7) areas. Additionally, based on previous literature (24), the baseline comorbidity for each person included hypertension (ICD-9-CM 401–405), stroke (ICD-9-CM 430–438), diabetes (ICD-9-CM 250), heart disease (ICD-9-CM 410–429), chronic kidney disease (CKD) (ICD-9-CM 585), tobacco use (ICD-9-CM 305.1), alcohol dependence syndrome (ICD-9-CM 303), and cancer (ICD-9-CM 140–208). Medication usage was identified based on whether the enrollees took corticosteroids or disease-modifying antirheumatic drugs for more than 6 months after the index date.

### Statistical Analysis

Firstly, the chi-square test and Student's *t*-test were employed to examine the baseline differences in sociodemographic characteristics and comorbidities between two groups. The Cox proportional hazards regression analysis was then applied to compute the adjusted hazard ratio (HR) with 95% confidence interval (CI) of dementia risk in association with RS use, after accounting for confounders reported at baseline, such as the demographic characteristics and comorbidities. To further test the robustness of the association of RS use with dementia risk, we categorized RS use as low intensity (1–3 courses), medium intensity (4–14 courses), or high intensity (≧15 courses) based on the former rule (16). A stratified analysis by age and sex using Cox proportional hazards regression was also conducted to assess the relative risk of dementia between the persons who did and did not receive RS. We also used time-dependent analysis with Cox proportional hazards regression to investigate the risk of dementia in relation to the time-dependent effect of RS. Additionally, log(-log[survival]) vs. log of survival time plot was inspected to verify the proportional hazards assumption. All analyses were conducted using SAS version 9.3 (SAS Institute Inc., Cary, NC, USA) and a *p*-value below 0.05 was considered statistically significant.

## Results

The study population consisted of 2,927 patients with RA (69.4% women) with a mean ± standard deviation age of 49.9 2 ± 11.62 years. Among them, 1,962 patients did not receive RS and 965 did. Compared to non-RS users, those receiving RS were more likely to be female, have heart disease, and reside in an urban area (all *p* < 0.05) (Table 1).

**Table 1:** Demographic characteristics and comorbidities of persons with RA who did and did not use RS.

**Variable**	**Non-RS users**	**RS users**	***P***
	**(*n* = 1962)**	**(*n* = 965)**	
Age*	49.57 (12.16)	50.11 (10.56)	0.24
Age			0.25
≤ 50	986 (50.3)	463 (48.0)	
>50	976 (49.7)	502 (52.0)	
Sex			<0.001
Female	1300 (66.3)	731 (75.8)	
Male	662 (33.7)	234 (24.2)	
Monthly income			0.38
Low	806 (41.1)	372 (38.5)	
Median	1062 (54.1)	541 (56.1)	
High	94 (4.8)	52 (5.4)	
Residential area			0.001
Urban	1113 (56.7)	605 (62.7)	
Suburban	320 (16.3)	136 (14.1)	
Rural	529 (27.0)	224 (23.2)	
Corticosteroid use			0.96
Yes	1682 (85.7)	828 (85.8)	
No	280 (14.3)	137 (14.2)	
Comorbidity			
Hypertension	1059 (54.0)	551 (57.1)	0.11
Stroke	383 (19.5)	212 (22.0)	0.12
Diabetes	663 (33.8)	347 (36.0)	0.25
Heart disease	962 (49.0)	527 (54.6)	0.01
Chronic kidney disease	164 (8.4)	74 (7.7)	0.52
Cancer	280 (14.3)	137 (14.2)	0.96
Alcohol dependence syndrome	18 (0.9)	3 (0.3)	0.07
Tobacco use	43 (2.2)	22 (2.3)	0.88

*Numbers indicate n (%) except for mean age*.

* *Indicating mean value (standard deviation)*.

Among all eligible patients with RA, a total of 1,662 first episodes of dementia occurred, including 1,224 among non-RS users and 388 among RS users, during the follow-up periods of 10604.53 and 5141.98 person-years (PY), respectively. The incidence rate of dementia was lower among RS users than in non-RS users (75.46 vs. 115.42 per 1,000 PYs), and the adjusted HR was 0.60 (95% CI = 0.53–0.67). Moreover, RA patients who used the high intensity of RS had a 62% lower risk of dementia (95% CI = 0.22–0.70) (Table 2). Additionally, the RA severity indices may affect this finding, but the severity data were not available in the LHID. To address this concern, we undertook two sensitivity analyses to verify the relationship between RS use and subsequent dementia risk. The first sensitivity analysis, limited to those with no comorbidities, found that RS was still protective against the development of dementia (adjusted HR = 0.57, 95% CI = 0.39–0.71). Second, we used the prescription of biological agents as a surrogate for RA severity, dividing them by whether or not they received biological agents for ≥6 months after the index date. The proportion of use of biological agents was 61.3% (592/965) in the RS user cohort and 60.8% (1,193/1,962) in the non-RS user cohort. After considering the influence of use of biological agents, RS use was still significantly related to the lower risk of dementia, with the adjusted HR of 0.56 (95% CI = 0.34–0.76). Accordingly, we inferred that disease severity did not appreciably confound the relationship obtained in this study.

**Table 2:** Crude and adjusted HR for dementia in RA patients who received or did not receive RS.

**Patient group**	**Events**	**PYs**	Incidence*	**HR (95% CI)**	***P***	Adjusted HR** (95% CI)	***P***
Non-RS users	1,224	10604.53	115.42	1		1	
RS users	388	5141.98	75.46	0.64 (0.57–0.71)	<0.001	0.60 (0.53–0.67)	<0.001
Low intensity	337	4185.72	80.51	0.69 (0.60–0.76)	<0.001	0.64 (0.57–0.73)	<0.001
Medium intensity	43	794.88	54.10	0.47 (0.35–0.64)	<0.001	0.42 (0.31–0.57)	<0.001
High intensity	8	161.39	49.57	0.43 (0.23–0.72)	0.003	0.38 (0.22–0.70)	0.005

* *Incidence rate is per 1,000 PYs*.

** *Model adjusted for age, sex, urbanization level, monthly income, corticosteroid usage, and comorbidities*.

Table 3 presents the analytic results stratified by age and sex. Collectively, the use of RS was associated with a lower risk of dementia, irrespective of sex. The multivariable stratified analysis further verified a significant association of lower risk of dementia with RS use among those 50 years and older (aHR = 0.56, 95% CI = 0.50–0.68). Table 4 indicated that the RS use provided the greatest benefit when initiated within the first 2 years after RA onset (adjusted HR = 0.38, 95% CI = 0.30–0.48).

**Table 3:** Incidence and dementia risk for RA patients with and without RS stratified by sex and age.

**Variables**	**Non-RS users**			**RS users**			**Crude HR (95% CI)**	***P***	**Adjusted HR (95% CI)**	***P***
	**Case**	**PYs**	**Incidence**	**Case**	**PY**	**Incidence**				
**Sex**										
Female	821	6886.45	119.22	300	3953.11	75.89	0.65 (0.55–0.72)	<0.001	0.61^γ^ (0.53–0.68)	<0.001
Male	403	3718.67	108.39	88	1188.87	74.02	0.67 (0.51–0.75)	<0.001	0.59^γ^ (0.47–0.73)	<0.001
**Age (years)**										
≤ 50	540	6102.57	88.49	153	2585.02	59.19	0.68 (0.53–0.77)	<0.001	0.67* (0.51–0.74)	<0.001
>50	684	4501.95	151.93	235	2556.96	91.92	0.59 (0.52–0.71)	<0.001	0.56* (0.50–0.68)	<0.001

Y *Model adjusted for age, urbanization level, monthly income, corticosteroid usage, and comorbidities*.

* *Model adjusted for sex, urbanization level, monthly income, corticosteroid usage, and comorbidities*.

**Table 4:** Risk of dementia in RA patients who used RS relative to those who did not use RS by length of follow up.

**Follow-up**	**Non-RS users**		**RS users**		
	**Adjusted HR**	**95% CI**	Adjusted HR*	**95% CI**	***P***
<2 years	1	Reference	0.38	0.30–0.48	<0.001
2–5 years	1	Reference	0.43	0.34–0.50	<0.001
5–10 years	1	Reference	0.64	0.48–0.81	<0.001
>10 years	1	Reference	0.86	0.72–0.95	0.003

* *Model adjusted for age, sex, urbanization level, monthly income, corticosteroid usage, and comorbidities*.

## Discussion

Given that RS is becoming more frequently an integral part of a multidisciplinary approach to RA management, it may be imperative to determine whether the RS use can lessen the predisposition of dementia for those affected by RA.

Over the 15-years study period, we found a 40% lower risk of dementia in those receiving RS than in those not receiving RS. Notably, after the onset of RA, RS use with the high intensity decreased the risk of developing dementia even further to 62%. Examination of the dose-response relationship may help in clarifying the nature of the causation between RS usage and the subsequent dementia risk. Despite the paucity of findings regarding the long-term impact of RS on sequent dementia risk among individuals with rheumatic disorders, this positive therapeutic effect observed with use of RS appeared to concurred with earlier reports and further adds to the literature on this topic (9, 11–13).

Several explanations have been proposed to explain the positive effect of RS against the dementia onset in RA patients. First, participation in physical activity training has been proven to inhibit the production of inflammatory cytokines in the body, such as interleukin (IL)-6, IL-1, and tumor necrosis factor-α (25). Reducing inflammatory markers can not only mitigate the uncomfortable symptoms of RA but also benefit synaptic and neuronal integrity, which in turn lessens the individual's susceptibility to cognitive impairment disorders. Recent evidence demonstrated that the above-mentioned inflammatory markers might play pivotal roles in the development or pathology of neurodegenerative diseases (26, 27). Second, a previous study also found the activation of intracellular signaling pathways, especially brain-derived neurotrophic factor (BDNF)—tyrosine receptor kinase B (TrkB), may account for the positive link between RS utilization and lower risk of neurological disorders (28). Upregulating the BDNF-TrkB pathway has been shown to rescue neuronal cells from neurodegeneration due to injuries in the central nervous system and to prevent further oxidative damage in cultivated neurons (29). Meanwhile, activation of BDNF-TrkB signaling would facilitate the long-term potentiation and formation of synapses, by triggering phosphorylation and the expression of proteins that are markers of synaptic plasticity, thus lessening the vulnerability to dementia (28).

In addition, findings from the present study revealed that older patients benefited most from RS by lowering the likelihood for dementia occurrence. We speculate that, compared to older adults, younger adults may have fewer coexisting medical conditions, better medical knowledge, better attitudes toward health, and are also more likely to comply with medical or health advices (30). Deficits in any of these areas could partially reduce the effect of RS on lowering dementia risk. Altogether, we recommend that an early inclusion of RS to routine pharmacological therapy, as well as prolonging its use, may serve to cognitively benefit patients with RA.

While our study is the first to investigate the relation between RS use and dementia risk among RA patients, there are several important limitations to consider. First, while using secondary health care databases, there could be careless errors in the coding process. To minimize this bias, we enrolled only persons with new-onset RA or dementia, and only after the patients had at least three outpatient visits reporting consistent diagnoses or at least one inpatient admission. It should also be noted that the NHI of Taiwan randomly reviewed the charts and audited medical charges to verify the accuracy of claim files (17). Furthermore, the coding approach and data availability were similar between the two groups, and the misclassification bias could have likely been non-differential and toward the null hypothesis, thus possibly inducing the underestimation of observed estimate. Second, the LHID lacks information on social network relationships, family history, laboratory data or educational level. Thus, the study linking administrative data and the abovementioned factors is worthy of further investigation. Third, the used database lacks a reliable severity index of RA, and failure to adjust for this factor may affect the findings. Nevertheless, we attempted to adjust for the possible comorbidities via the use of multivariate statistical analysis of the data and the prescription of biological agents to avoid confounding as much as possible. Findings from the two sensitivity analyses supported the present finding that the RA severity did not mislead the conclusion reported herein. Fourth, we could not shed further light on the corresponding effect of specific RS modalities on dementia risk because the fees regarding RS intervention during healthcare procedures were integrated and presented in one consolidated file of the NHIRD. Fifth, the stratification of RS intensity was made arbitrarily, so the causal relationships implied by our findings should be interpreted with caution. A randomized controlled trial is recommended to better determine the efficacy of RS, as well as to uncover the mechanisms underpinning the positive impact of RS. These limitations notwithstanding, this study also had considerable advantages. The database applied in this study is representative of the entire Taiwanese population, and this large sample size ensured obtaining reliable findings. In addition, the application of longitudinal cohort study design could robustly examine the causal relation between the high intensity of RS use and lower risk of dementia among RA subjects.

## Conclusion

This study explored the possibility that the use of RS may lessen the sequent risk of dementia among the individuals with RA, which could be a reference for further studies regarding RS influence on other medical complications due to RA. Although the findings from the current study indicated that use of RS by RA groups was related to lower dementia risk, future prospective randomized trials that overcome the limitations of this study are warranted to provide more conclusive evidence of the association suggested in this study.

## Data Availability Statement

The datasets analyzed in this article are not publicly available. Data are available from the National Health Insurance Research Database (NHIRD) published by Taiwan National Health Insurance (NHI) Bureau. Due to legal restrictions imposed by the government of Taiwan in relation to the “Personal Information Protection Act,” data cannot be made publicly available. Requests to access the datasets should be directed to the NHIRD and the corresponding authors Ning-Sheng Lai (q12015@tzuchi.com.tw) or Tzung-Yi Tsai (dm732024@tzuchi.com.tw).

## Ethics Statement

The studies involving human participants were reviewed and approved by Institutional Review Board and Ethics Committee of Buddhist Dalin Tzu Chi Hospital, Taiwan (No. B10004021-3). Written informed consent for participation was not required for this study in accordance with the national legislation and the institutional requirements.

## Author Contributions

HL, H-LH, S-WY, and T-YT: study concept and design. M-CLu, N-SL, and T-YT: acquisition of data. HL, T-YT, M-CLi, and C-TY: data analysis. N-SL, M-CLu, and T-YT: project management. M-CLi, H-LH, HL, T-YT, C-TY, S-WY, and N-SL: writing. All authors contributed to the article and approved the submitted version.

## Conflict of Interest

The authors declare that the research was conducted in the absence of any commercial or financial relationships that could be construed as a potential conflict of interest.
